# Factors Associated With Difficult Emergency Department Referral: A Cross-Sectional Study

**DOI:** 10.7759/cureus.99516

**Published:** 2025-12-18

**Authors:** Abdulrahman A Aldhibaib, Yahia Y Akeely, Taleb S Almarri, Husam A Alkhathlan, Alya A Alshammari, Swaid R Saulat, Mohammed A Alseghair

**Affiliations:** 1 Emergency Department, Security Forces Hospitals Program, General Directorate of Medical Services, Ministry of Interior, Riyadh, SAU; 2 Emergency Department, College of Medicine, Dar Al-Uloom University, Riyadh, SAU

**Keywords:** challenges, consultations, difficulties, emergency department, referrals

## Abstract

Background: Emergency department (ED) referrals are an essential component of patients’ care. According to the reviewed literature, ED referrals to other specialties frequently encounter difficulties. Nonetheless, the specific challenges of the Saudi context have not received adequate attention.

Methods: A cross-sectional study of emergency physicians in Riyadh City was conducted through a questionnaire using Google Forms. We gathered data on sociodemographic, referral practices, awareness, and difficulties. Data were analyzed using descriptive statistics and chi-square tests, with a significance level of 0.05.

Results: Of the 104 participants, the majority (94, 90.4%) reported difficulty with referrals. The most common reasons for referral difficulties are the following: 86 (82.7%) report that consultants are being asked to refer patients to other specialties before seeing them in person, 79 (76.0%) refuse referrals due to "endorsement time," and 76 (73.1%) request further tests. Unexpectedly, 62 (59.6%) of the participants reported verbal assault. On the other hand, there was a strong relationship between gender and referral difficulties, with 27 (100%) of female physicians reporting challenges versus 67 (87%) of male physicians (p=0.049). In addition, 39 (37.5%) study participants had no formal training in effective referrals. We also noticed that the majority of the difficulties occurred during the night and weekend shifts (61, 58.7%, and 54, 51.9%, respectively).

Conclusion: The most obvious reasons are consulting physicians ordering additional labs or images, or asking to refer the patient to another specialty before seeing them. In addition, consulting physician endorsement time was an issue. Furthermore, referral difficulties occur more frequently during night shifts and weekends. To mitigate these difficulties, all training programs should include communication and training skills. Policies of consultation or referral should be enforced. Additionally, the emergency physician should be able to schedule outpatient appointments with the required specialty. Moreover, the receiving consultant should be a senior physician. Referral difficulties should be minimized to improve patient care, outcomes, and satisfaction.

## Introduction

Emergency department (ED) referrals are a fundamental component in patients' care [1]. In North America, referral rates in the emergency department range from 20% to 40% [2,3]. ED is frequently referred to as the "front door" of a hospital, a place of organized chaos where the primary goal is to stabilize, diagnose, and discharge patients. Referral and consultation when an emergency physician seeks input or formal patient transfer to a specialist or admitting team is an important step in this process. This seemingly simple step is actually one of the most complex aspects of emergency medicine. It encounters difficulties, resulting in a significant delay in patient flow.

The delay in accepting referrals from emergency physicians will lead to overcrowding. This will have a negative impact on the patients’ care and experience [4]. Specialist consultation is a primary contributor to increased ED length of stay (LOS), accounting for 33%-54% of the total time a patient spends in the department [5-7]. Such delays will have negative consequences for the patient's health outcomes [8].

Effective patient management in the emergency department necessitates the collaboration of physicians from various specialties. This is due to the fact that most emergency cases present with multiple concurrent medical and surgical complaints. These cases require urgent attention and management. Any conflict in this process will result in unnecessary delays [9].

Furthermore, inappropriate communication and referral difficulties will affect not only the patient but also the emergency physician’s mental and physical well-being. In addition, these difficulties may lead to depression, loss of interest, and quitting work among the emergency physicians [10].

The existing literature provides different studies that address these challenges and potential solutions [4,11]. Nevertheless, these challenges and solutions differ between practicing hospitals in various countries. Given this context, a study should be conducted locally to identify specific causes and potential solutions. Moreover, to our knowledge, no prior research in Saudi Arabia has examined these challenges.

These challenges, which include negotiating ambiguous admission criteria, obtaining timely acceptance from specialists, and managing patients' and their families' expectations during delays, are daily sources of stress that contribute to physician burnout and emergency department overcrowding. This study will thus focus on the important aspect of referral acceptance in relation to the preceding difficulties, with the ultimate goal of reducing the strain and stress that emergency physicians face while improving the care that patients receive.

The objectives of this study are to identify the presence of these difficulties. Moreover, the study aims to elucidate all potential causes and strive towards a feasible solution. The hypothesis of this study is that all difficulties faced in referrals in the emergency department will affect patients' care and safety.

## Materials and methods

Study design and location

This paper is a descriptive, cross-sectional study. It was done using Google Forms. It followed the STrengthening the Reporting of Observational Studies in Epidemiology (STROBE) guidelines for observational studies. The study included emergency physicians from a variety of hospitals in Riyadh, Saudi Arabia, including public, private, military, and academic institutions. The data were gathered between June and August 2025.

Population and sample for the study

This study includes all licensed emergency physicians, residents, and specialists. To qualify for the study, participants were required to have a minimum of one year of experience working in an emergency department prior to enrollment. Physicians practicing outside the emergency department or related specialties were excluded. The target population comprises 149 emergency physicians employed at four tertiary care hospitals within Riyadh City. Successfully, we received 104 participants out of 149, corresponding to a response rate of 69.8%.

Tools and methods for collecting data

A Google Form was used to gather the data. The questionnaire was developed and went through a full validation process with a pilot study. Based on the feedback, all of the survey questions have been changed to make them clearer and more accurate. The questionnaire was sent out electronically through professional social media groups, email lists, and direct personal contacts to ensure that as many people as possible in the target population got it.

There were four main parts in the questionnaire. Part A: Demographic and professional data, such as age, gender, years of experience, and job title (Appendix, Table 6). Part B: All possible reasons for difficult ED referrals (Appendix, Table 7). Part C: The possible measures to reduce these difficulties (Appendix, Table 8).

Analyzing data

We used Statistical Product and Service Solutions (SPSS, version 23.0; IBM SPSS Statistics for Windows, Armonk, NY) to extract and analyze the data we collected. For categorical variables, descriptive statistics were presented as frequencies and percentages. For continuous variables, means and standard deviations or medians and interquartile ranges were used to summarize the data, depending on how normal the distribution was. Chi-square tests were used to look at the links between demographic variables and how people thought about consultation difficulties. A p-value under 0.05 was considered statistically important.

Ethical considerations

Ethical approval for this study was obtained from the Institutional Review Board (IRB) of the Security Forces Hospital’s scientific committee with this number (KACST, KSA: H-01-R-069). Participation was entirely voluntary and anonymous. An informed consent statement was included on the first page of the electronic questionnaire. All data were kept confidential. All data were kept confidential and used only for the purposes of this research.

## Results

This study included a total of 104 physicians. Table 1 shows the socio-demographic profile of the participants. There were 52 (50%) between 25 and 30 years, 19 (18.3%) were between 31 and 35 years, 10 (9.6%) were between 36 and 40 years, seven (6.7%) were between 41 and 45 years, and 16 (15.4%) were older than 45 years. As for the gender, 77 (74%) were males, while 27 (26%) were females.

**Table 1:** Socio-demographic profile of the participants (n=104)

Age in years	Number		%
20-30 years		52	50.0
31-35 years		19	18.3
36-40 years		10	9.6
41-45 years		7	6.7
Older than 45 years		16	15.4
Gender			
Male		77	74
Female		27	26
Total		100	100%

Table 2 presents the career profiles of the participants. Regarding the level of training, five (4.8%) were general practitioners, 52 (50%) were training residents, nine (8.7%) were registrars, and 38 (36.5%) were senior registrars. As for the experience, 61 (58.6%) had 0-5 years of experience, 14 (13.5%) had 6-10 years of experience, and 29 (27.9%) had more than 10 years of experience. Regarding the board at which the physician is currently training or from which they have graduated, 83 (79.8%) reported the Saudi board, 16 (15.4%) reported a non-Saudi board, and five (4.8%) reported not having a training board.

**Table 2:** Career profile of the participants (n=104)

Level	Number	%
General practitioner	5	4.8
Training resident	52	50.0
Registrar	9	8.7
Senior registrar	38	36.5
Experience		
0-5 years	61	58.6
6-10 years	14	13.5
More than 10 years	29	27.9
Which training Board are you currently training at or graduated from?		
Saudi Board	83	79.8
Non-Saudi Board	16	15.4
No training Board	5	4.8
Total	104	100

**Figure 1:**
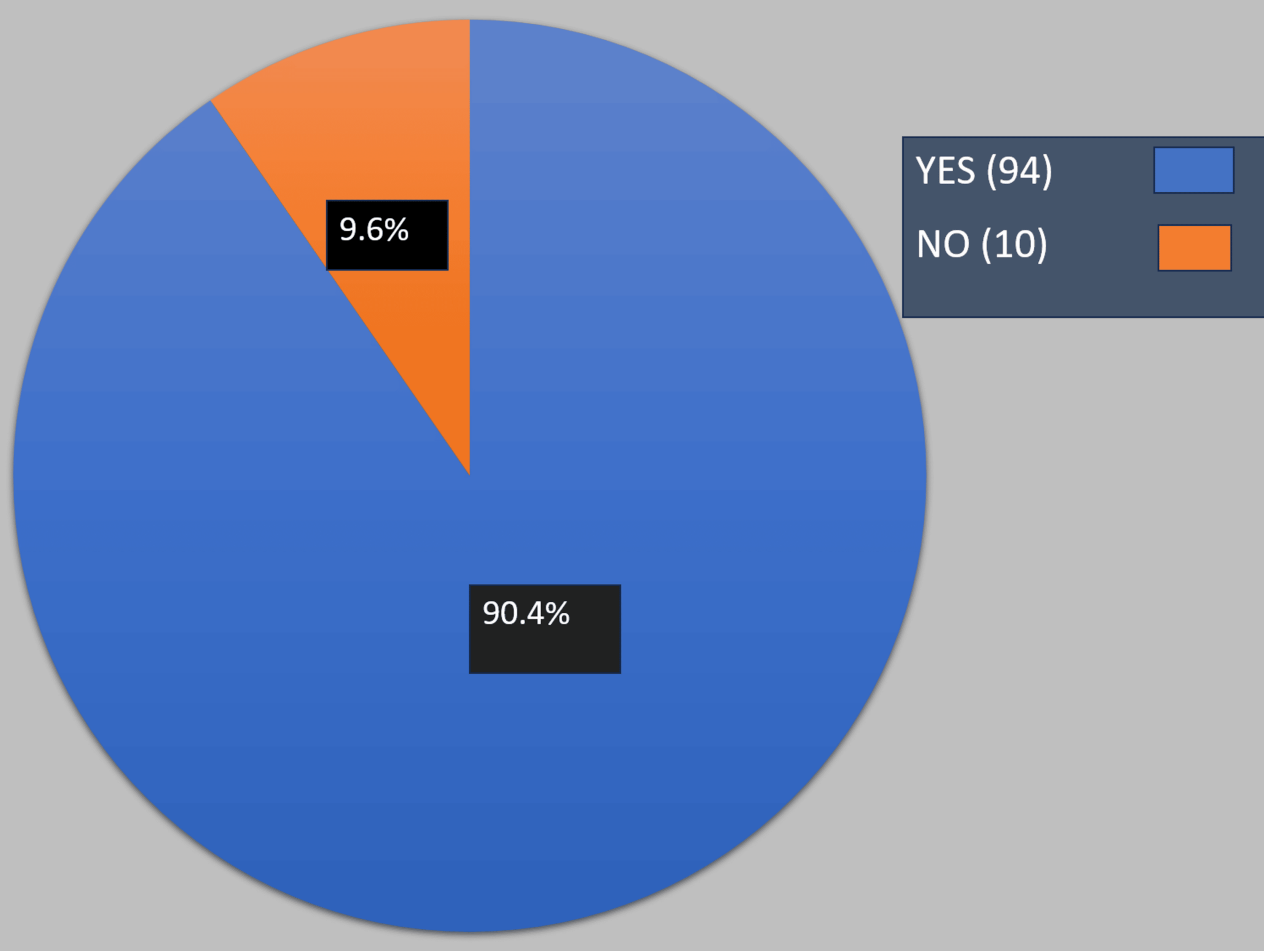
Results of the question, "Do you believe that there are difficulties during ED referrals?"

The participants were asked, "Do you believe that there are difficulties during referrals?" Ninety-four participants (90.4%) believed there were difficulties during referrals, while 10 (9.6%) did not, as shown in Figure 1.

Table 3 demonstrates the participants' experiences and practices regarding referrals. Regarding the frequency of making referrals on a shift basis, 59 (56.7%) participants reported referring 0-5 patients per shift, 37 (35.6%) reported referring 6-10 patients per shift, and eight (7.7%) reported referring more than 10 patients per shift. Lacking access to outpatient booking was a common reason for referral for 88 (84.6%) participants. Regarding the time of the week that was most challenging, 50 (48.1%) participants reported working during the week, while 54 (51.9%) reported working on weekends. Among the physicians, 62 (60.0%) reported experiencing a verbal assault by a consulting physician, one (1%) reported experiencing both a verbal and physical assault, and 41 (39%) reported not experiencing any form of assault. Furthermore, 63 (60.6%) of the respondents reported that they discuss cases with their consultant or team leader before referring them to other specialties. Regarding who is more resistant to receiving referrals, 19 (18.3%) reported that seniors are resistant, 55 (52.9%) reported that juniors are resistant, and 30 (28.8%) reported that both groups are resistant. Regarding training, 65 participants (62.5%) reported that they received training on how to conduct an effective referral.

**Table 3:** Participants experience and practices with referrals (n=104)

Question	Number	%
How often do you refer patients on a shift bases		
0-5 patients per shift	59	56.7
6-10 patients er shift	37	35.6
More than 10 patients per shift	8	7.7
Have you ever referred a patient in the ED because you lack access to outpatient booking		
Yes	88	84.6
No	16	15.4
Which time of the week is the most challenging?		
Working days	50	48.1
Weekends	54	51.9
Have you ever experienced any form of assault, whether verbal or physical, by a consulting physician		
Yes, verbally	62	60.0
Yes, both verbally and physically	1	1.0
No	41	39.0
Do you discuss with your consultant/team leader before you refer a case to other specialties		
Yes	63	60.6
No	9	8.7
Not applicable	32	30.7
Which level is more resistant to receive referrals		
Seniors	19	18.3
Juniors	55	52.9
Both	30	28.8
Have you ever received a training on conducting effective Referral?		
Yes	65	62.5
No	39	37.5
What challenges are you likely to face during the referral process? (You can choose more than one option)		
The on-call physician is asking to refer the patient to other specialties (before assessing the patient)	86	82.7
The on-call physician is refusing the referral due to the endorsement time	79	75.7
The on-call physician is requesting further investigations (before assessing the patient)	76	73.1
The on-call physician is refusing to see the patient	66	63.5
The on-call physician is not responding	54	51.9
The patient is too complicated and needs multiple teams	52	50.0
The on-call physician has communication issues	44	42.3
The on-call physician is giving orders over the phone (before assessing the patient)	40	38.5
No clear policy on which specialty should receive the referral	38	36.5
Not reaching a final diagnosis makes the referral more difficult	37	35.6

When asked about the challenges that physicians are likely to face during the referral process, the most commonly reported issues included the on-call physician requesting to refer the patient to another specialty before assessing the patient (reported by 86 participants, or 82.7%); the on-call physician refusing the referral due to endorsement time (reported by 79 participants, or 75.7%); and the on-call physician requesting further investigations before assessing the patient (reported by 76 participants, or 73.1%).

**Figure 2:**
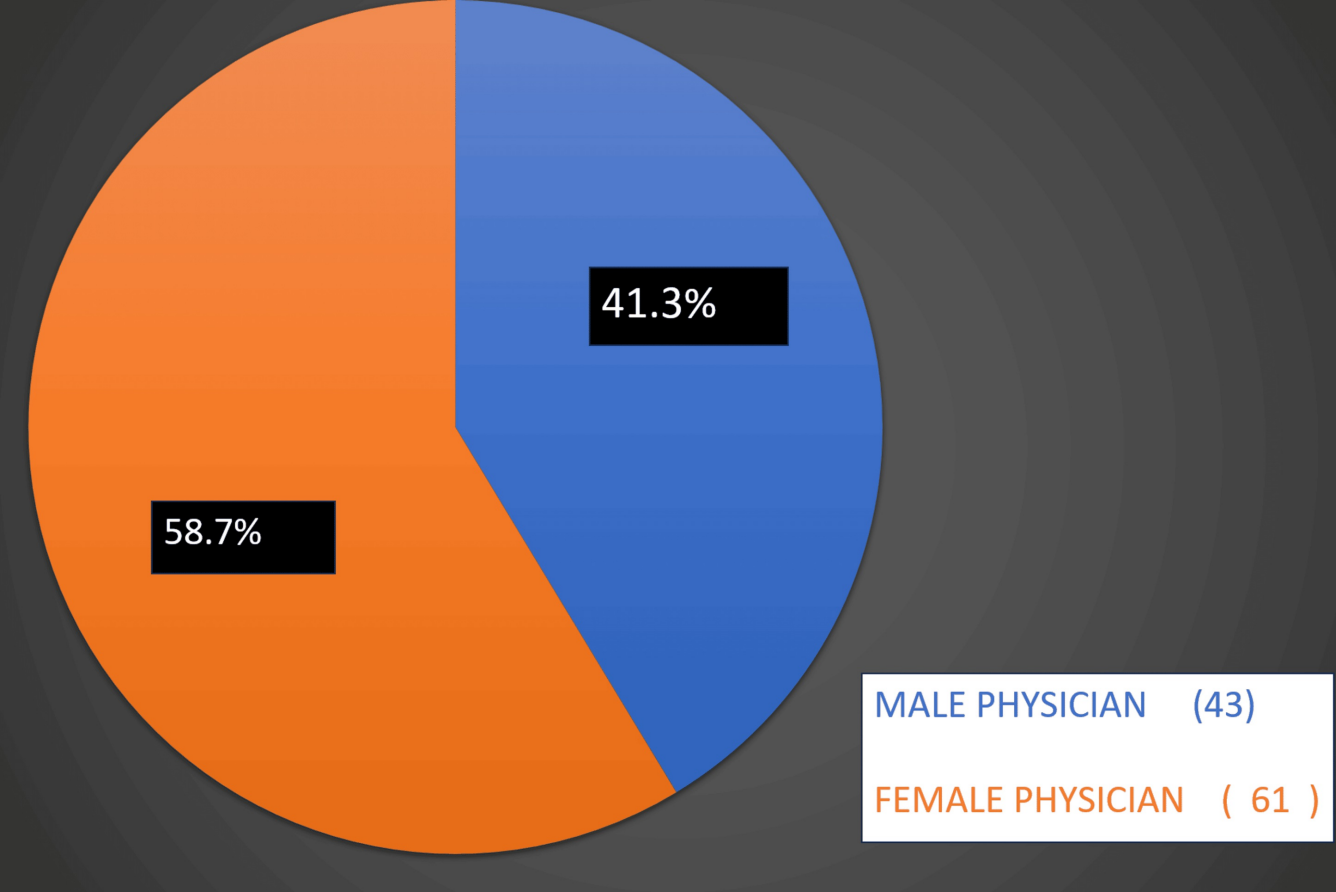
Results to the question, "In your opinion, which gender is more difficult to receive referrals?"

Figure 2 illustrates the participants' answers to "In your opinion, which gender is more difficult to receive referrals?"; 43 (41.3%) reported males being more difficult, while 61 (58.7%) reported females being more difficult.

**Figure 3:**
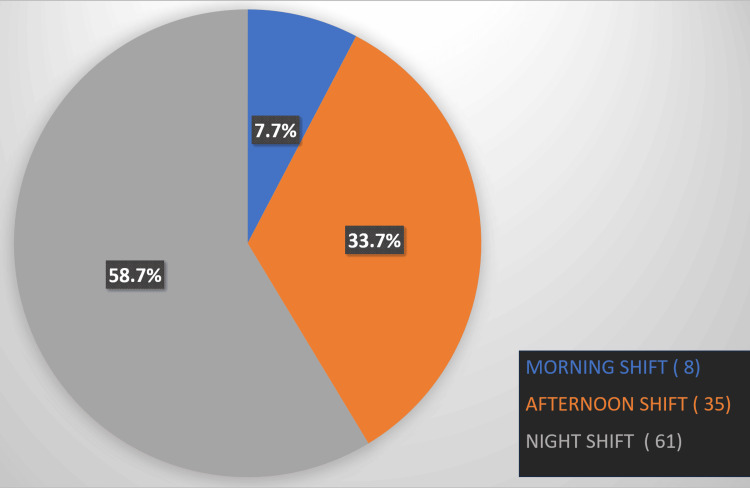
Results to the question, "In your opinion, which shift timing is more difficult to make a referral?"

Figure 3 illustrates the participants' responses to the question, "In your opinion, which shift timing is more difficult to make a referral?" There were eight (7.7%) reported morning shifts, 35 (33.7%) reported afternoon shifts, and 61 (58.7%) reported night shifts.

Table 4 displays the referral policies implemented in the department. Most of the participants (102, 98.1%) reported that their hospital has a policy for ED referrals, while two (1.9%) reported that their hospital does not have such a policy. Furthermore, 73 (70.2%) of the participants reported that the on-call physicians usually know the hospital policy, while 31 (29.8%) reported that the on-call physicians usually do not know it. Additionally, 99 (95.2%) of the participants reported that they are aware of and adhere to the hospital policy for ED referrals. Furthermore, 95 (91.3%) of the respondents reported that their hospital has a policy for resolving and escalating conflicts, while nine (8.7%) indicated that their hospital does not have such a policy.

**Table 4:** Referral policies in the department (n=104)

Question	Number	%
Does your hospital have a policy for ED referrals		
Yes	102	98.1
No	2	1.9
Does the on-call physician usually knows and follows the hospital policy		
Yes	73	70.2
No	31	29.8
Do you know and follow the hospital policy for ED referral		
Yes	99	95.2
No	3	2.9
My department does not have a referral policy	2	1.9
Does your hospital have a policy for solving and escalating conflicts		
Yes	95	91.3
No	9	8.7

Table 5 presents the factors associated with the belief that difficulties occur during referrals. Females were found to have a significantly higher rate of believing that there are difficulties during referrals compared to males, with 27 (100%) of females and 67 (87%) of males expressing this belief, resulting in a p-value of 0.049. However, the factors of age, level, years of experience, training board, and frequency of referrals did not show a correlation with the belief that difficulties exist during referrals.

**Table 5:** Factors associated with believing that there is difficulties during referrals

Factor	Believing that there are difficulties during referrals		P-value
	Yes	No	
Age in years			0.842
25-30 years	47 (90.4%)	5 (9.6%)	
31-35 years	17 (89.5%)	2 (10.5%)	
36-40 years	10 (100%)	0 (0%)	
41-45 years	6 (85.7%)	1 (14.3%)	
More than 45 years	14 (87.5%)	2 (12.5%)	
Gender			0.049*
Male	67 (87%)	10 (13%)	
Female	27 (100%)	0 (0%)	
Level			0.638
General practitioner	4 (80%)	1 (20%)	
Training resident	46 (88.5%)	6 (11.5%)	
Registrar	8 (88.9%)	1 (11.1%)	
Senior registrar	36 (94.7%)	2 (5.3%)	
Experience			0.745
0-5 years	54 (88.5%)	7 (11.5%)	
6-10 years	13 (92.3%)	1 (7.1%)	
More than 10 years	27 (93.1%)	2 (6.9%)	
Which Board are you currently training at or graduated from?			0.661
Saudi Board	75 (90.4%)	8 (9.6%)	
Non-Saudi Board	15 (93.8%)	1 (6.3%)	
No training board	4 (80%)	1 (20%)	
How often do you refer patients on a shift bases			0.228
0-5 patients per shift	53 (89.8%)	6 (10.2%)	
6-10 patients per shift	35 (94.6%)	2 (5.4%)	
More than 10 patients per shift	6 (75%)	2 (25%)	
*Significant at level 0.05			

## Discussion

In this study, we highlighted the obvious burden of these difficulties. Most of the participants agreed about the existence of significant challenges during referrals. Regarding the important reasons for referral difficulties, we noticed the following: the consultant physician asked to refer the patient to another specialty before the assessment, refused referral because of "endorsement time,” and asked for further tests. Our findings are consistent with those of Eshikumo, who similarly identified refusal to see patients and ambiguous responsibility as significant sources of conflict [9].

The concerning fact that over 50% of physicians reported experiencing verbal assaults indicates that such conflicts frequently result in unprofessional conduct, representing a significant issue in the study of workplace behavior. These findings corroborate the research conducted by Ariza-Montes et al. and Benning et al. [12,13].

A notable finding is the correlation between gender and the perception of challenges. All of the female emergency physicians reported difficulties encountered during their referral process, while 87% of the male emergency physicians did, with a significant p-value (p=0.049). This suggests that female physicians may face additional, gender-specific challenges or biases in inter-physician communication, a complex area necessitating further qualitative investigation. No previous studies of a similar nature have noted this finding. On the other hand, regarding the effect of timing, the majority of referrals were perceived as more challenging during night shifts and weekends, which corresponds with diminished staffing and resource availability, a recognized factor contributing to ED crowding. This finding is similar to previous studies done by Hajzargarbashi et al. and Mostafa et al. [14,15].

Moreover, the prevalent practice of referring patients due to inadequate outpatient booking access suggests that EDs are functioning as alternatives to overloaded primary and specialty care systems, a significant factor contributing to non-emergent referrals and crowding, as noted by Greenwood-Lee et al. [16]. Furthermore, it indicates the lack of ED physicians’ privileges to arrange an outpatient appointment.

Although almost everyone claims to have formal ED referral policies, the challenges with referrals remain significant. In spite of that, the difficulties in referrals, as shown in this study, are significantly greater. This "policy-practice gap" is an important finding because it shows that policies alone are not enough; they need strong implementation, monitoring, training, and accountability systems to work. This disconnect may help explain why conflicts are still common, even though people are very aware of the policies.

Another compelling finding is that our research revealed a deficiency in training. This deficiency in formal education regarding a fundamental professional competency represents a significant modifiable factor. It corroborates the recommendations of various studies advocating for the incorporation of standardized consultation frameworks, such as the 5-C model or PIQUED, into residency training and continuous professional development [17-19].

In our study, we discovered that people perceived junior physicians as the most resistant to referrals. This resistance is due to their insufficient knowledge and experience. For this finding, the consulting physician should be a senior resident or registrar. This individual will make a quick decision, plan, and early disposition in contrast to the junior resident receiving the referrals [20].

Strengths and limitations

In this study, the targeted population consisted of individuals from various large hospitals. It includes military, private, government, and academic hospitals. The questionnaire was developed through proper validation tests and assessment. The questions cover all aspects of the referral difficulties. It covers the size of these difficulties, awareness, possible reasons, and solutions. On the other hand, there are different limitations to this study. First, it is an observational study, so it cannot demonstrate causation. It provides general suggestions, as well as possible causes of referral difficulties. Second, doing it in various Saudi cities and regions would make it more general. Third, it would be better if other medical services had been included, such as internal medicine or general surgery, to explore their views. It will provide useful information for specific solutions. As a result, we believe that now is an excellent time to conduct research that addresses this limitation. Finally, there were no open-ended questions. It could elaborate more on difficulties and solutions.

## Conclusions

There are different reasons for difficulties in referring patients in the ED. It includes a lack of training, inappropriate communications, and unprofessional behavior. Furthermore, there are insufficient available appointments for outpatient follow-up. Night shifts and weekends are the most difficult times for referrals. These times should be investigated, and a root cause analysis should be conducted. Further strategies to reduce such difficulties are training programs that must incorporate communication skills and strategies for managing referral and consultation difficulties into their teaching curriculum. The ED physician should be privileged to conduct an outpatient follow-up. Furthermore, a senior resident or registrar should receive the referral and consultation for better decisions and time-saving. Improving the referral process is crucial for enhancing patient care and safety.

## Appendices

**Table 6:** Demographic and professional data

Age	-
Gender	-
Job title	-
Years of experience	-
Name the hospital you are working at	-
Which training board did you graduate from, or are you currently studying at?	-

**Table 7:** Possible reasons for difficult ED referrals

Do you believe that there are difficulties during referrals?	-
How often do you refer patients on a shift basis?	-
Have you ever referred a patient in the ED because you lack the access to outpatient booking?	-
Which time of the week is the most challenging?	-
Which gender is most difficult to receive referrals?	-
Which shift timing makes it more difficult to make referrals?	-
It is a difficult referral because the on-call physicians refuse to see the patient.	-
It is a difficult referral because the on-call physicians said it is the endorsement time.	-
It is a difficult referral because the on-call physicians ask for more investigations/images before seeing the patient.	-
It is a difficult referral because the on-call physicians ask to refer the case to another specialty.	-
It is a difficult referral because the on-call physicians are not responding to the call.	-
It is a difficult referral because the on-call physicians have communication difficulties and negative attitudes.	-
It is a difficult referral because the case itself is complicated and needs different specialties at the same time.	-
It is a difficult referral because there is no clear policy about the admission pathway that indicates which specialty should admit the patient.	-
It is a difficult referral because there is no final diagnosis yet for the patient.	-

**Table 8:** Possible measures to reduce ED referral difficulties

Do you discuss the case with your team leader before referral to other specialties?	-
Does your hospital have a policy for ED referrals?	-
Does the on-call physician usually know and follow the hospital policy?	-
Have you ever experienced any form of assault during referral?	-
Which level has more resistance to receiving referrals?	-
Have you ever received training on conducting effective referrals?	-
Do you know and follow the hospital policy for ED referral?	-
Does your hospital have a policy for resolving conflicts in referral?	-
